# *MYBL2* Gene Polymorphism Is Associated With Acute Lymphoblastic Leukemia Susceptibility in Children

**DOI:** 10.3389/fonc.2021.734588

**Published:** 2021-09-08

**Authors:** Haixia Guo, Na Li, Yaping Sun, Cuiling Wu, Huixia Deng, Ling Xu, Xu Yang

**Affiliations:** ^1^Department of Pediatrics, Nanfang Hospital, Southern Medical University, Guangzhou, China; ^2^Institute of Systems Biology, Shenzhen Bay Laboratory, Shenzhen, China; ^3^Institute of Synthetic Biology, Shenzhen Institutes of Advanced Technology, Chinese Academy of Sciences, Shenzhen, China; ^4^Department of Hematology, Guangzhou Women and Children’s Medical Center, Guangzhou Medical University, Guangzhou, China

**Keywords:** acute lymphoblastic leukemia (ALL), **MYBL2**, single nucleotide polymorphisms (SNPs), susceptibility, children, IKZF1

## Abstract

**Purpose:**

Although *MYBL2* had been validated to participate in multiple cancers including leukemia, the role of **MYBL2** polymorphisms in acute lymphoblastic leukemia (ALL) was still not clear. In this study, we aimed to evaluate the association between **MYBL2** single nucleotide polymorphisms (SNPs) and ALL risk in children.

**Methods:**

A total of 687 pediatric ALL cases and 971 cancer-free controls from two hospitals in South China were recruited. A case-control study by genotyping three SNPs in the **MYBL2** gene (rs285162 C>T, rs285207 A>C, and rs2070235 A>G) was conducted. The associations were assessed by odds ratios (ORs) with corresponding 95% confidence intervals (CIs). Subgroup and stratification analyses were conducted to explore the association of rs285207 with ALL risk in terms of age, sex, immunophenotype, risk level, and other clinical characteristics. The false-positive report probability (FPRP) analysis was performed to verify each significant finding. Functional analysis in silico was used to evaluate the probability that rs285207 might influence the regulation of **MYBL2**.

**Results:**

Our study demonstrated that rs285207 was related to a decreased ALL risk (adjusted OR = 0.78; 95% CI = 0.63-0.97, *P* = 0.022) in the dominant model. The associations of rs285207 with ALL risk appeared stronger in patients with pre B ALL (adjusted OR=0.56; 95% CI=0.38-0.84, *P*=0.004), with normal diploid (adjusted OR=0.73; 95% CI=0.57-0.95, *P*=0.017), with low risk (adjusted OR=0.68; 95% CI=0.49-0.94, *P*=0.021), with lower WBC (adjusted OR=0.62; 95% CI=0.43-0.87, *P*=0.007) or lower platelet level (adjusted OR=0.76; 95% CI=0.59-0.96, *P*=0.023). With FPRP analysis, the significant association between the rs285207 polymorphism and decreased ALL risk was still noteworthy (FPRP=0.128). Functional analysis showed that IKZF1 bound to DNA motif overlapping rs285207 and had a higher preference for the risk allele A. As for rs285162 C>T and rs2070235 A>G, no significant was found between them and ALL risk.

**Conclusion:**

In this study, we revealed that rs285207 polymorphism decreased the ALL risk in children, and rs285207 might alter the binding to IKZF1, which indicated that the **MYBL2** gene polymorphism might be a potential biomarker of childhood ALL.

## Introduction

Acute lymphoblastic leukemia (ALL) is a malignant proliferation of poorly differentiated lymphocytes, the most common type of pediatric leukemia and also the most frequently diagnosed malignancy in children (1, 2). ALL is still one of the most important causes of childhood morbidity and mortality, despite medicine development and elevated cure rates in the last decades (3). The etiology of ALL is still not well clear, although mechanisms involved in it have been extensively investigated. In addition to environmental factors, gene etiology remains the predominant pathogenesis of childhood ALL (3, 4). Single-nucleotide polymorphisms (SNPs) in cancer-associated key genes (*CDKN2A*, *GATA3*, *FOXO3, etc.*) had been reported to influence ALL risk in children (5–7). However, many of the variations in genes influencing ALL remain to be found.

**MYBL2** (MYB proto-oncogene like 2), also known as B-MYB, belongs to the MYB family of transcription factor which was first identified as a vertebrate homolog of the v-myb oncogene causing leukemia in chickens (8, 9). The MYB transcription factor family included MYBL1 (A-MYB), *MYBL2* (B-MYB), and MYB (c-MYB) (10, 11). However, unlike the other two members which are usually expressed in certain tissues, *MYBL2* is ubiquitously expressed in proliferating cells (12, 13). **MYBL2** has also been demonstrated to act as an oncogene in numerous studies (14, 15). *MYBL2* promotes the malignant development of cancer *via* regulating various cellular processes including apoptosis, proliferation, differentiation, invasion, metastasis, and replication stress (12, 16–20). The aberrant expression or dysfunction of *MYBL2* has been validated in a variety of cancers including adult leukemia, breast cancer, prostate cancer, ovarian cancer, liver cancer, and lung cancer (19–25). However, the association of **MYBL2** gene polymorphisms with childhood ALL risk and outcomes has not been reported. In the current study, we explored the association of **MYBL2** SNPs with ALL risk among a case-control series of Chinese children.

## Materials and Methods

### Study Subject

In total, 687 ALL patients and 971 healthy controls were included in the current study. All the individuals were enrolled from Guangzhou Women and Children’s Medical Center and Nanfang Hospital. Briefly, ALL patients < 18 years old confirmed with a clinical and histological diagnosis were recruited. All patients were newly diagnosed, and a detailed medical history was recorded for each case. Thus, 687 children with ALL were recruited from January 2016 to June 2019. During the same period, 971 cancer-free healthy volunteers were also collected as controls, which were matched to the cases on age, sex, and residential region. The controls were randomly selected from children undergoing a routine physical examination. All included subjects were ethnic Han Chinese. In addition, those with other malignant disorders, a history of chemotherapy or radiotherapy were excluded. The written informed consent was obtained from each subject before participation. This study obtained permission from the institutional review boards of both hospitals.

### SNP Selection and Genotyping

The SNP selection was performed using data from SNPinfo and NCBI dbSNP databases, and the selection strategy was based on four criteria as described previously (26, 27): (1) the minor allele frequency (MAF) reported in HapMap was >5% for Chinese subjects; (2) located in or near the *MYBL2* gene (i.e., < 2kb upstream or downstream of *MYBL2*); (3) affecting transcription factor binding sites (TFBS) activity, splicing activity or protein-coding; (4) not in high linkage disequilibrium (LD, R^2^ < 0.8). Based on the above criteria, three SNPs (rs285162 C>T, rs285207 A>C, and rs2070235 A>G) in **MYBL2** gene were retrieved for further analyses.

Peripheral blood samples were collected from each participant at diagnosis and then used for DNA extraction using the TIANamp DNAKit (TianGen, Beijing, China) according to the manufacturer’s instruction. rs285162, rs285207 and rs2070235 were selected for genotyping. In the genotyping assays, the Taqman ProAmp master mix and pre-designed SNP genotyping assay mix containing polymerase chain reaction (PCR) probes and primers (ABI, Massachusetts, USA) were used. The quantitative real-time PCR method was performed to genotype these three SNPs using QuantStudio™ 6 Flex System (ABI, Massachusetts, USA). In each of the 384-well plates, five positive and five negative controls were included to ensure the accuracy of genotyping. About 10% of samples were selected randomly for direct sequencing to ensure quality control (28, 29), and the results were 100% concordant.

### Functional Analysis *In Silico*

The probability that rs285207 A>C might influence the regulation of **MYBL2** was evaluated by using the Roadmap Epigenome Browser (30, 31), TFBIND software (32), and the ENCODE Project (33). Briefly, promoter and enhancer were predicted *via* histone modification and DNase hypersensitivity (DHS) of GM12878 (lymphocyte cell line) in Roadmap Epigenomics data. TFBIND was used to assess whether rs285207 altered any transcription factor binding sites (TFBS), and then ENCODE ChIP-seq experiments of IKZF1 in GM12878 (Experiment Series: ENCSR816OIY) was used to assess the binding signals and motifs overlapping rs285207.

### Statistical Analyses

For each SNP in controls, Hardy-Weinberg equilibrium (HWE) was assessed *via* the goodness-of-fit χ^2^ test. Genotype distribution of each SNP and demographic variables between the case and control group was analyzed using a 2-sided χ^2^ test. To evaluate the strength of the relation between **MYBL2** polymorphisms and ALL susceptibility, odds ratios (ORs) and 95% confidence interval (95% CIs) were calculated using logistic regression analyses, adjusting for age and sex. The false-positive report probability (FPRP) was also computed for each significant finding as previously described (34). A prior probability of 0.1 was adopted to detect an OR of 0.67 for protective effects, and an FPRP value reaching the threshold of <0.2 was considered noteworthy. All statistical analyses were conducted with SAS software (version 9.4; SAS Institute, Cary, North Carolina). In this study, all *P* values were 2-sided, and a *P* value of <0.05 was considered as statistical significance.

## Results

### Subject Characteristics

In the present study, a total of 687 cases and 971 controls were included, and the detailed characteristics are summarized in **Table 1**. Briefly, no significant differences were observed in age (*P* =0.494) or sex (*P* =0.107) distribution between cases and controls. According to immunophenotype-based classification (35–37), 596 (86.75%) cases were diagnosed with B cell ALL (B ALL), including 227 (33.04%) pro B ALL, 200 (29.11%) common B ALL, 166 (24.16%) pre B ALL, and 3 (0.44%) mature B ALL; 61 (8.88%) diagnosed with T cell ALL (T ALL); 30 (4.37%) with no available data (NA). Besides, information about gene infusion, risk level, karyotype, relapse, and the levels of minimum residual disease (MRD) at multiple time points post-therapy were also included in **Table 1**.

**Table 1:** Frequency distribution of selected characteristics in ALL cases and cancer-free controls.

Variables	ALL Cases (n=687)		Controls (n=971)		*P* ^a^
	No.	%	No.	%	
Age range, years	0.67-17		0.92-15		0.494
Mean ± SD	5.04 ± 3.02		5.44 ± 2.78		
<10	609	88.65	871	89.70	
≥10	78	11.35	100	10.30	
Sex					0.107
Female	278	40.47	355	36.56	
Male	409	59.53	616	63.44	
Immunophenotype					
B ALL	596	86.75			
Pro B	227	33.04			
Common B	200	29.11			
Pre B	166	24.16			
Mature B	3	0.44			
T ALL	61	8.88			
NA	30	4.37			
Gene fusion type					
BCR-ABL	18	2.62			
ETV6-RUNX1	125	18.20			
E2A-PBX1	21	3.06			
SIL-TAL	7	1.02			
MLL	11	1.60			
Other fusions	14	2.04			
Normal with no fusion	483	70.31			
NA	8	1.16			
Risk level					
Low	240	34.93			
Medium	319	46.43			
High	62	9.02			
NA	66	9.61			
Karyotype					
Hypo-diploid	19	2.77			
Normal diploid	441	64.19			
Abnormal diploid	39	5.68			
Low hyper-diploid	18	2.62			
High hyper-diploid	56	8.15			
NA	114	16.59			
MRD in marrow(%, 19d)					
<0.01	7	1.02			
≥0.01	384	55.90			
NA	296	43.08			
MRD in marrow(%, 35d)					
<0.01	237	34.50			
≥0.01	205	29.84			
NA	245	35.66			
MRD in marrow(%, 12w)					
<0.01	290	42.21			
≥0.01	33	4.81			
NA	364	52.98			
Relapse					
−	465	67.69			
+	19	2.77			
NA	203	29.54			

SD, standard deviation; NA, not available; MRD, minimum residual disease;

a Two-sided χ^2^ test for distributions between ALL cases and cancer-free controls.

### Associations Between **MYBL2** Gene Polymorphisms and ALL Susceptibility

According to the SNP selection strategy, three SNPs (rs285162 C>T, rs285207 A>C, and rs2070235 A>G) that overlapped with transcription factor binding site (TFBS), splicing regulating site (SRS), or non-synonymous SNP (nsSNP) were selected (**Table 2**). The genotype frequencies of **MYBL2** gene SNPs in all 687 cases and 971 controls and their association with ALL risk were described in **Table 3**. All these three SNPs were in HWE (*P*_HWE_ *>*0.05) among the controls. Of the three SNPs, significant differences were observed for rs285207 A>C (crude *P* = 0.017) between ALL cases and controls in a dominant model. After adjustment with age and sex, rs285207 C allele was significantly related to a decreased ALL risk in the dominant model (AC/CC vs AA: adjusted OR = 0.78; 95% CI = 0.63-0.97, *P* = 0.022). The rest two genotypes (rs285162 C>T and rs2070235 A>G), however, were not significantly associated with ALL risk.

**Table 2:** SNPs captured by the three selected *MYBL2* polymorphisms as predicted by SNPinfo software (http://snpinfo.niehs.nih.gov/).

rs number	Chr	Allele	TFBS	SRS	nsSNP	Genomic position (GRCh37)	Allele	Asian	CHB
rs285162	20	C/T	–	Y	–	42328639	C	0.881	0.887
rs285207	20	A/C	Y	–	–	42295379	A	0.742	0.774
rs2070235	20	A/G	–	–	Y	42328639	A	0.853	0.892

SNP, single nucleotide polymorphism; TFBS, transcription factor binding sites; SRS, splicing regulating site; CHB, Han Chinese in Beijing, China.

**Table 3:** Logistic regression analysis of associations between *MYBL2* polymorphisms and ALL susceptibility.

Genotype	Cases (N=687)	Controls (N=971)	*P* ^a^	Crude OR (95% CI)	*P*	Adjusted OR (95% CI) b	*P* ^b^
rs285162 (HWE=0.7301)							
CC	553 (82.41)	796 (83.35)		1.00		1.00	
CT	111 (16.54)	152 (15.92)		1.05 (0.80-1.37)	0.715	1.06 (0.81-1.38)	0.697
TT	7(1.04)	7 (0.73)		1.44 (0.50-4.12)	0.498	1.48 (0.51-4.26)	0.467
Additive			0.541	1.08 (0.85-1.37)	0.541	1.08 (0.85-1.38)	0.514
Dominant	118 (83.35)	159 (16.65)	0.621	1.07 (0.82-1.39)	0.620	1.07 (0.83-1.40)	0.597
Recessive	664 (98.96)	948 (99.27)	0.505	1.43 (0.50-4.09)	0.507	1.47 (0.51-4.22)	0.477
rs285207 (HWE=0.0797)							
AA	502 (73.18)	656 (67.70)		1.00		1.00	
AC	165 (24.05)	292 (30.13)		**0.74 (0.59-0.92)**	**0.008**	**0.75 (0.60-0.94)**	**0.011**
CC	19 (2.77)	21 (2.17)		1.18 (0.63-2.22)	0.603	1.18 (0.62-2.22)	0.616
Additive			0.059	0.83 (0.69-1.01)	0.059	0.84 (0.69-1.02)	0.072
Dominant	184 (26.82)	313 (32.30)	**0.017**	**0.77 (0.62-0.95)**	**0.017**	**0.78 (0.63-0.97)**	**0.022**
Recessive	667 (97.23)	948 (97.83)	0.432	1.29 (0.69-2.41)	0.433	1.27 (0.68-2.39)	0.452
rs2070235 (HWE=0.0527)							
AA	577 (84.73)	798 (82.18)		1.00		1.00	
AG	99 (14.54)	170 (17.51)		0.81 (0.62-1.06)	0.116	0.81 (0.62-1.07)	0.133
GG	5 (0.73)	3 (0.31)		2.31 (0.55-9.68)	0.254	2.33 (0.55-9.81)	0.250
Additive			0.278	0.87 (0.67-1.12)	0.278	0.87 (0.68-1.13)	0.309
Dominant	104 (15.27)	173 (17.82)	0.173	0.83 (0.64-1.08)	0.173	0.84 (0.64-1.10)	0.196
Recessive	676 (99.27)	968 (99.69)	0.220	2.39 (0.57-10.0)	0.235	2.41 (0.57-10.1)	0.232

a χ^2^ test for genotype distributions between ALL cases and cancer-free controls.

b Adjusted for age and sex.

The bold values were statistically significant results.

### Subgroup and Stratification Analyses

To further explore the association between the **MYBL2** gene rs285207 A>C polymorphism and ALL susceptibility, subgroup and stratification analyses were performed in terms of age, sex, karyotype, immunophenotype, gene infusion, risk level, and other clinical information. All the results were shown in **Table 4**. Compared with the rs285207 AA genotype, the AC/CC genotype decreased ALL risk in females (adjusted OR=0.68; 95% CI=0.47-0.97, *P*=0.033), and children < 10 years old (adjusted OR=0.78; 95% CI=0.62-0.98, *P*=0.030). For the immunophenotype, the rs285207 AC/CC genotype decreased ALL risk in patients with B ALL (adjusted OR=0.78; 95% CI=0.62-0.98, *P*=0.035), particularly in patients with pre B ALL (adjusted OR=0.56; 95% CI=0.38-0.84, *P*=0.004). The rs285207 AC/CC genotype also decreased ALL risk in children with normal diploid (adjusted OR=0.73; 95% CI=0.57-0.95, *P*=0.017), with low risk (adjusted OR=0.68; 95% CI=0.49-0.94, *P*=0.021), with lower WBC (adjusted OR=0.62; 95% CI=0.43-0.87, *P*=0.007) or lower platelet level (adjusted OR=0.76; 95% CI=0.59-0.96, *P*=0.023). Furthermore, the rs285207 AC/CC genotype reduced ALL risk in patients without relapse (adjusted OR=0.78; 95% CI=0.61-0.99, *P*=0.046). The level of MRD is a key prognosis factor for ALL patients, and the negative MRD (<0.01%) usually indicates a good prognosis. In this study, MRD was also analyzed according to different therapy strategies (**Table S1**). In patients treated with China Children’s Cancer Group (CCCG)-ALL-2015 protocol, no association was observed between MRD level and rs285207 polymorphism. In the group of South China Children Leukemia Group (SCCLG)-ALL-2016 protocol, the rs285207 AC/CC genotype seemed to increase the rates of patients with negative MRD (<0.01%) (adjusted OR= 3.10, 2.67 and 999 at day 19, day 35 and week 12, respectively), although no significant result was found due to the limited sample size.

**Table 4:** Subgroup and stratification analysis of *MYBL2* polymorphisms with ALL susceptibility.

Variables	rs285207 (cases/controls)			Adjusted OR^a^	*P* ^a^
	AA	AC/CC		(95% CI)	
Age, years					
<10	444/588	164/281		**0.78 (0.62-0.98)**	**0.030**
≥10	58/68	20/32		0.73 (0.37-1.41)	0.341
Sex					
Females	212/243	66/112		**0.68 (0.47-0.97)**	**0.033**
Males	290/413	118/201		0.84 (0.64-1.10)	0.210
Immunophenotyping					
B ALL	435/656	160/313		**0.78 (0.62-0.98)**	**0.035**
Pro B	166/656	61/313		0.79 (0.57-1.09)	0.156
Common B	135/656	64/313		1.01 (0.73-1.40)	0.949
Pre B	131/656	35/313		**0.56 (0.38-0.84)**	**0.004**
Mature B	3/656	0/313		0.001(0.001-999)	0.950
T ALL	45/656	16/313		0.73 (0.40-1.31)	0.289
Gene fusion type					
BCR-ABL	11/656	7/313		1.25 (0.48-3.31)	0.648
ETV6-RUNX1	101/656	24/313		**0.51 (0.32-0.81)**	**0.004**
E2A-PBX1	15/656	6/313		0.85 (0.33-2.22)	0.741
SIL-TAL	6/656	1/313		0.34 (0.04-2.83)	0.318
MLL	8/656	3/313		0.80 (0.21-3.03)	0.738
Others	10/656	4/313		0.84 (0.26-2.71)	0.776
Normal	347/656	135/313		0.83 (0.65-1.05)	0.125
Risk level					
Low	183/656	57/313		**0.68 (0.49-0.94)**	**0.021**
Medium	225/656	93/313		0.87 (0.66-1.15)	0.339
High	47/656	15/313		0.67 (0.37-1.22)	0.191
Karyotype					
Normal diploid	327/656	113/313		**0.73 (0.57-0.95)**	**0.017**
Abnormal diploid	27/656	12/313		0.93 (0.46-1.86)	0.837
Hypo-diploid	11/656	8/313		1.47 (0.58-3.70)	0.415
Low hyperdiploid	12/656	6/313		1.05 (0.39-2.82)	0.928
High hyperdiploid	38/656	18/313		1.03 (0.57-1.84)	0.930
WBC					
Lower	166/656	48/313		**0.62 (0.43-0.87)**	**0.007**
Higher	181/656	75/313		0.87 (0.65-1.18)	0.382
Normal	102/656	45/313		0.95 (0.65-1.38)	0.774
Hemoglobin					
Lower	384/656	149/313		0.82 (0.65-1.04)	0.105
Higher	2/656	0/313		0.001 (0.00-999)	0.946
Normal	60/656	17/313		0.59 (0.34-1.04)	0.066
Platelet					
Lower	366/656		131/313	**0.76 (0.59-0.96)**	**0.023**
Higher	5/656		6/313	2.54 (0.77-8.40)	0.126
Normal	79/656		30/313	0.80 (0.52-1.25)	0.327
Relapse					
−	340/656		125/313	**0.78 (0.61-0.99)**	**0.046**
+	13/656		6/313	0.96 (0.36-2.55)	0.937

a Adjusted for age and sex.

Normal, values within reference range; Lower, values less than the lower limit of reference range; Higher, values higher than the upper limit of the reference range; The reference range of WBC (10^9^/L): 5-12; The reference range of Platelet (10^9^/L): 140-440.

The bold values were statistically significant results.

The FPRP values for these remarkable results at different levels of prior probability and statistical power were shown in **Table 5**. In the FPRP analysis, all the statistically significant findings were noteworthy at the prior probability level of 0.25 (FPRP<0.200). At the prior probability of 0.1, the association between the rs285207 AC/CC genotype and decreased ALL risk was still noteworthy (FPRP=0.128, statistical power=0.903), especially in patients with pre B ALL (FPRP=0.191, statistical power=0.469), normal diploid (FPRP=0.190, statistical power=0.738), lower WBC (FPRP=0.135, statistical power=0.327) or lower platelet level (FPRP=0.183, statistical power=0.855), which further strengthen the significant results above.

**Table 5:** False-positive report probability analysis for the significant findings.

Genotype	Crude OR (95% CI)	*P* ^a^	Statistical power^b^	Prior probability				
				0.25	0.1	0.01	0.001	0.0001
rs285207 A > C								
AC/CC vs. AA	0.77 (0.62-0.95)	0.015	0.903	**0.047**	**0.128**	0.618	0.942	0.994
Age <10 years	0.78 (0.62-0.98)	0.033	0.904	**0.098**	0.247	0.783	0.973	0.997
Female	0.68 (0.47-0.97)	0.033	0.533	**0.158**	0.360	0.861	0.984	0.998
Pre B ALL	0.56 (0.38-0.84)	0.005	0.469	**0.073**	**0.191**	0.722	0.963	0.996
TEL-AML fusion	0.51 (0.32-0.81)	0.004	0.124	**0.095**	0.240	0.776	0.972	0.997
Normal diploid	0.73 (0.57-0.95)	0.019	0.738	**0.072**	**0.190**	0.720	0.963	0.999
Low risk	0.68 (0.49-0.94)	0.019	0.536	**0.099**	0.247	0.783	0.973	0.996
Lower WBC	0.62 (0.43-0.87)	0.006	0.327	**0.050**	**0.135**	0.632	0.946	0.994
Lower platelet	0.76 (0.59-0.96)	0.021	0.855	**0.070**	**0.183**	0.712	0.961	0.996
No relapse	0.78 (0.61-0.99)	0.041	0.894	**0.121**	0.293	0.820	0.979	0.998

a Chi-square test was used to calculate the genotype frequency distributions.

b Statistical power was calculated using the number of observations in the subgroup and the OR and P values in this table.

The bold values were statistically significant results.

### Functional Analysis

**Figure 1:**
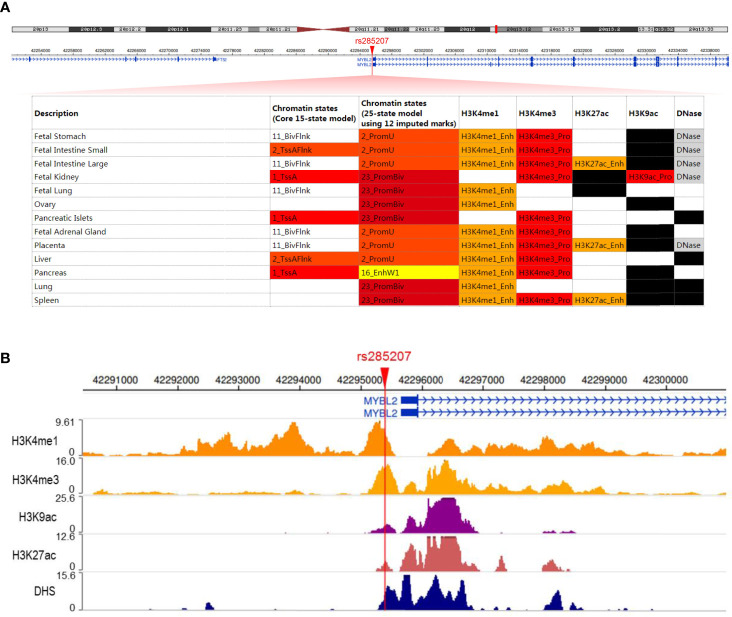
rs285207 overlapped both promoter and enhancer of *MYBL2* gene **(A)** Schematic of rs285207 region with histone and DHS mark annotations in different tissue types in the Roadmap epigenomics data. **(B)** H3K4me1, H3K4me3, H3K9ac, H3K27ac and DHS ChIP-seq signals at the rs285207 locus in GM12878 cells.

**Figure 2:**
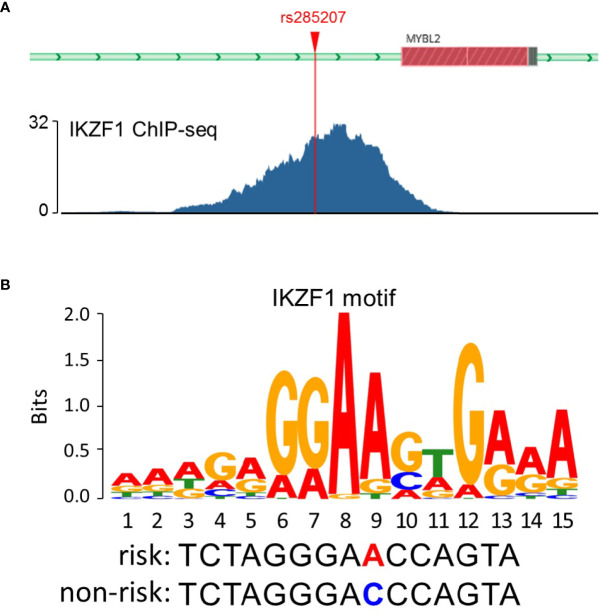
rs285207 modulated the binding to IKZF1. **(A)** IKZF1 ChIP-seq signals at the rs285207 locus in GM12878 cells. **(B)** Predicted preferential binding of IKZF1 to the risk allele A of rs285207.

To explore the potential mechanisms by which rs285207 influences the ALL risk, we evaluated the probability of rs285207 polymorphism altering transcription regulation of **MYBL2**. The Roadmap Epigenomics data showed that rs285207 overlapped DHS marks and histone modifications related to both promoter and enhancer in multiple tissue types (**Figure 1A**). This observation was further supported by H3K4me1, H3K4me3, H3K9ac, H3K27ac and DHS ChIP data in GM12878 cells (**Figure 1B**). TFBIND analysis revealed that rs285207 altered the binding affinity of this site to transcription factors including IKZF1, THAP4 and FOXA2. For the rs285207 risk allele A, all of IKZF1, THAP4 and FOXA2 have high TFbind scores (0.85, 0.77 and 0.86, respectively), while none of these three bindings was found for the non-risk allele C. In ENCODE ChIP-seq analysis, however, only IKZF1 was found to bind to DNA motif overlapping rs285207 (**Figure 2A**). IKZF1 has a higher preference for the risk allele A and leave no chance for allele C (**Figure 2B**). The results showed that rs285207 A>C might influence the transcription of **MYBL2* via* disrupting IKZF1 binding site.

## Discussion

In this case-control study with 687 ALL cases and 971 healthy controls from a Chinese population, we investigated the potential association between **MYBL2** gene polymorphisms and ALL risk in children. Among these three SNPs of **MYBL2** in this study, we found that rs285207 A>C was significantly associated with a decreased ALL risk in the dominant model. To our knowledge, the present study is the first to explore the association between **MYBL2** polymorphisms and ALL risk.

*MYBL2* is a member of the MYB transcription factor family which including MYBL1, *MYBL2*, and MYB (10, 11). Products encoded by the MYB family have similar protein structures: a DNA-binding domain, a transactivation domain, and a regulatory domain (9, 38). All members of the MYB family exert their action through regulating transcription of target genes by binding to the same DNA consensus sequence (NAACNG) (8, 39, 40). However, only *MYBL2* is ubiquitously expressed in proliferating cells (12, 13). The expression of **MYBL2** gene is controlled by other transcription factors or noncoding RNA (41–45), and the *MYBL2* protein is activated *via* phosphorylation (14, 21). After activated, *MYBL2* regulates downstream diverse genes or proteins involved in multiple cellular processes, such as *BCL2* and *MYC* in cell survival (46–48), cyclins and FGF4 in cell cycle (49–51), SOX2 and OCT4 in cell differentiation (52, 53), SNAIL and YAP1 in cell invasion and metastasis (19, 54). Overexpression or amplification of **MYBL2** had been widely reported in previous studies on cancer. For instance, *MYBL2* was overexpressed in castration-resistant prostate cancer and promoted cell growth and metastatic by promoting YAP1 transcriptional activity (19). Liang *et al.* reported that **MYBL2** expression was increased in gallbladder cancer and could serve as a potential prognostic biomarker (55). In addition, the **MYBL2** polymorphisms were reported to be associated with cancer (39, 56, 57). For example, Thorner *et al.* demonstrated that rs2070235 polymorphism was related to the increased risk of basal-like breast cancer (39). To date, there is no study detecting the polymorphisms of **MYBL2** gene in ALL.

In the present study, we performed genotyping of three potential functional SNP sites (rs285162, rs285207, and rs2070235). We found that the rs285207 polymorphism was associated with the reduced ALL risk, for the first time. Besides, in the subgroup analysis, the significant association between rs285207 and ALL patients with lower platelet level was achieved with a high statistical power (>0.8), which further strengthened the significant results above. Rs285207 is located in the 374bp upstream of **MYBL2** gene, which overlaps with the **MYBL2** promoter and enhancer. In our analyses of transcription factor binding, rs285207 A>C was also found to disrupt the binding to IKZF1 which showed preferential binding of the risk allele A. IKZF1, also called IKAROS, belongs to the transcription factor family of zinc-finger proteins (58). *IKZF1* played a key role in lymphopoiesis and also was a predisposition gene of leukemia (59–61). Therefore, rs285207 might influence the transcription of **MYBL2* via* altering the affinity of its binding to IKZF1. The effects of rs285207 polymorphism on ALL risk might be achieved by controlling the expression of **MYBL2** gene, which needs to be validated in future studies. The rest two SNPs, rs285162 and rs2070235, are both located in the code region of **MYBL2**. Rs285162, with the potential to regulate splicing, was reported to influence the deviation between allele frequencies (62). Rs2070235, which results in a missense variant (Ser > Gly) of **MYBL2** protein, was reported to be associated with an increased risk of breast cancer (39), but not with the incidence of acute myeloid leukemia (63). Besides, rs2070235 was found to be related to the reduced cancer risk in an investigation integrating several cancers (neuroblastoma, colon carcinoma, and chronic myelogenous leukemia) (64). In the current study, neither rs285162 nor rs2070235 was found to be associated with childhood ALL susceptibility. Along with previous reports, this study suggests that the **MYBL2** gene polymorphism is complex, depending on cancer types. In addition, the variety of ethnicity and sample composition should be taken into consideration.

Although the present study is the first to explore the relationship between **MYBL2** polymorphisms and ALL risk in children, several limitations should be acknowledged. First, only three SNPs of **MYBL2** were investigated in this study and more potentially functional SNPs need to be done in future studies. Second, although the sample size in this study was a relatively large one, studies with larger sizes should be conducted in the future. Third, the subjects were all retrieved from south China, which might cause selection bias, and therefore multicenter studies with more populations are needed to further confirm the role of **MYBL2** polymorphisms in ALL. Finally, this study just explored the genetic factor, but environmental factors including dietary intake were not available. The functions of **MYBL2** SNPs in the progression of ALL also need to be further investigated.

## Conclusion

In conclusion, the case-control study explored the association of *MYBL2* polymorphisms (rs285162, rs285207, and rs2070235) with childhood ALL risk and firstly demonstrated that the rs285207 A>C in **MYBL2** gene decreased the risk of childhood ALL and might influence the transcription of **MYBL2* via* altering IKZF1 binding, which suggested that **MYBL2** polymorphism might serve as a biomarker for ALL susceptibility. Certainly, larger multicenter-based studies and functional experiments are encouraged to further elucidate the role of **MYBL2** polymorphism and the potential mechanisms in ALL.

## Data Availability Statement

The original contributions presented in the study are included in the article/**Supplementary Material**, further inquiries can be directed to the corresponding author/s.

## Ethics Statement

The study was reviewed and approved by the institutional review boards of Guangzhou Women and Children’s Medical Center and Nanfang Hospital. Written informed consent to participate in this study was provided by the participants’ legal guardian/next of kin.

## Author Contributions

HG and XY designed the study. HG, NL, YS, and XY wrote the manuscript. NL, CW, HD, and LX treated the patients, collected the data, and commented on the manuscript. CW, HD, and LX performed the genotyping assay. YS and XY performed statistical analysis and function analysis. All authors contributed to the article and approved the submitted version.

## Funding

This study was supported by grants from National Natural Science Foundation of China (81870115, 32001043), Guangdong Basic and Applied Basic Research Foundation (2020A1515110886), and Guangzhou Municipal Science and Technology Project (202102020344).

## Conflict of Interest

The authors declare that the research was conducted in the absence of any commercial or financial relationships that could be construed as a potential conflict of interest.

## Publisher’s Note

All claims expressed in this article are solely those of the authors and do not necessarily represent those of their affiliated organizations, or those of the publisher, the editors and the reviewers. Any product that may be evaluated in this article, or claim that may be made by its manufacturer, is not guaranteed or endorsed by the publisher.
