# Supplementary material for: MYBL2 Gene Polymorphism Is Associated With Acute Lymphoblastic Leukemia Susceptibility in Children
Source: Front Oncol. 2021 Sep 8;11:734588. doi: 10.3389/fonc.2021.734588 (PMC8456030; doi:10.3389/fonc.2021.734588)
Supplement: Supplementary file 1 [file Table_1.docx]

| **Table S1.** The influence of MYBL2 polymorphisms (rs285207 , AC/CC vs. AA) on MRD levels based on different treatment strategies | | | | | | | | | | | | | |
| --- | --- | --- | --- | --- | --- | --- | --- | --- | --- | --- | --- | --- | --- |
| Treatment |  | MRD in marrow(%, 19d) | | | | MRD in marrow(%, 33d) | | | | MRD in marrow(%, 12w) | | | |
|  |  | Case (%) | | *P*^a^ | Adjusted OR ^a^  (95% CI) | Case (%) | | *P*^a^ | Adjusted OR ^a^  (95% CI) | Case (%) | | *P*^a^ | Adjusted OR ^a^  (95% CI) |
|  |  | <0.01 | ≥0.01 |  |  | <0.01 | ≥0.01 |  |  | <0.01 | ≥0.01 |  |  |
| CCCG-  ALL-2015 | AA | 5(2.08) | 235(97.92) |  | 1.00 | 153(52.58) | 138(47.42) |  | 1.00 | 185(92.04) | 16(7.96) |  | 1.00 |
|  | AC/CC | 0(0.00) | 96(100.00) | 0.983 | 0.001(0.00-999) | 59(50.00) | 59(50.00) | 0.633 | 0.90 (0.59-1.38) | 75(93.75) | 15(6.25) | 0.688 | 1.24 (0.44-3.52) |
| SCCLG-  ALL-2016 | AA | 1 (5.88) | 16(94.12) |  | 1.00 | 17(70.83) | 7(29.17) |  | 1.00 | 20(90.91) | 2(9.09) |  | 1.00 |
|  | AC/CC | 1(12.50) | 7(87.50) | 0.563 | 3.10 (0.07-144) | 8(88.89) | 1(11.11) | 0.419 | 2.67 (0.25-28.9) | 10(100.00) | 0(0.00) | 0.975 | 999 (0.00-999) |
| ^a^ Adjusted for age and gender, and refers to odd ratio of patients with negative MRD(<0.01%).  CCCG: Chinese Children Cancer Group; SCCLG: South China Children Leukemia Group; NA: Not Available. | | | | | | | | | | | | | |
